# Tuberculosis: The success tale of less explored dormant *Mycobacterium tuberculosis*

**DOI:** 10.3389/fcimb.2022.1079569

**Published:** 2022-12-22

**Authors:** Akanksha Verma, Antara Ghoshal, Ved Prakash Dwivedi, Ashima Bhaskar

**Affiliations:** Immunobiology Group, International Centre for Genetic Engineering and Biotechnology, New Delhi, India

**Keywords:** *Mycobacterium tuberculosis (M.tb)*, Tuberculosis (TB), LTBI, dormancy, resuscitation, vaccines, drugs, Host-directed therapies (HDT)

## Abstract

*Mycobacterium tuberculosis* (*M.tb*) is an intracellular pathogen that predominantly affects the alveolar macrophages in the respiratory tract. Upon infection, the activation of TLR2 and TLR4- mediated signaling pathways leads to lysosomal degradation of the bacteria. However, bacterium counteracts the host immune cells and utilizes them as a cellular niche for its survival. One distinctive mechanism of *M.tb* to limit the host stress responses such as hypoxia and nutrient starvation is induction of dormancy. As the environmental conditions become favorable, the bacteria resuscitate, resulting in a relapse of clinical symptoms. Different bacterial proteins play a critical role in maintaining the state of dormancy and resuscitation, namely, DevR (DosS), Hrp1, DATIN and RpfA-D, RipA, etc., respectively. Existing knowledge regarding the key proteins associated with dormancy and resuscitation can be employed to develop novel therapies. In this review we aim to highlight the current knowledge of bacterial progression from dormancy to resuscitation and the gaps in understanding the transition from dormant to active state. We have also focused on elucidating a few therapeutic strategies employed to prevent *M.tb* resuscitation.

## 1 Introduction

The second most common infectious killer and 13th leading cause of death worldwide, behind COVID-19, is tuberculosis (TB). In 2022, the WHO stated that there were over 1.5 million annual deaths, further emphasizing the need to eradicate TB. *Mycobacterium tuberculosis* (*M.tb*), initially identified by Dr. Robert Koch in 1882 (Cambau and Drancourt, 2014), is the cause of tuberculosis. *M.tb* is a slow-growing, acid-fast aerobic bacterium, with a doubling time of 18-24 hours (Smith, 2003; Beste et al., 2009). It is one of the oldest bacteria on the planet, with origin dating back to around 150 million years ago. *M.tb* has been known by diverse names such as *phthisis* in ancient Greece, *tabes* in ancient Rome, *schachepheth* in ancient Hebrew, *scrofula* in Middle Ages and *white plague* in the 1700s (Barberis et al., 2017). *M.tb* is a successful intracellular pathogen. When inhaled through aerosol droplets it affects primarily the lungs and can eventually disseminate to other organs such as the brain, liver, kidney, and pancreas. The pathogenesis of the bacterium has already been reviewed elsewhere (Smith, 2003; Bañuls et al., 2015; Ehrt et al., 2018). In a nutshell, the pathogenesis can be summed up as infection of the alveolar macrophages which phagocytose and attempt to eliminate the pathogen *via* lysosomal-dependent degradation of the phagosome containing the pathogen (Hmama et al., 2015). However, the bacteria have evolved various strategies to counteract the host defense systems. It inhibits the phagolysosome fusion and replicates within host macrophages, followed by slowing down to a low metabolic state called dormancy. The bacteria take advantage of the host factors and environment, due to which they remain in dormant state for years without showing clinical symptoms (Boom et al., 2021). Nonetheless, co-infections and other causes of immunosuppression can assist the bacteria to resuscitate and replicate thereby causing active TB and increasing the threat of transmission of *M.tb* to other healthy individuals.

To date, researchers are relentlessly trying to understand the exact mechanism of transition of bacteria from dormant to metabolically active state in the host. Battling the disease to enhance the safety of individuals requires a deep understanding of bacterial factors that utilize the host machinery to subvert host immune responses. The understanding of the twofold relationship between the host and bacteria can help design better therapeutics to augment existing anti-TB drugs.

Being a serious life-threatening disease, there are still major conundrums associated with TB. These include improper diagnosis of latent TB in endemic areas (Zellweger et al., 2020), prediction of reactivation in patients with latent infection, less efficient vaccine (the only available vaccine is BCG) (Brandt et al., 2002), and multi-drug resistant TB (MDR, XDR) (Singh et al., 2020; Hu et al., 2021). It is a pre-requisite to address the above-mentioned pitfalls to curtail TB transmission and eventually boost the WHO End TB campaign by 2035. To better understand the problems associated with latent TB and TB recurrence, in this review, we have highlighted the possible mechanisms and factors responsible for bacterial dormancy, persistence, and resuscitation. We have briefly summarised the existing models to study dormancy and resuscitation. We endeavour to underline the conventional and prospective novel targets of host and bacteria which can be employed to design an efficacious therapy against TB.

## 2 Dormancy, persistence, and models of dormancy

Dormancy in *M.tb* refers to the state of viability but metabolic inactivity under unfavorable physiological conditions and immune pressure faced in the host. Immunologically unfavorable conditions include challenges posed by various cells of innate and adaptive immune compartments *via* production of cytokines, chemokines, interleukins, increased phagocytosis, rapid antigen processing and presentation. Such stresses result in reduced metabolism to improve *M.tb* survival, but the bacteria can resume their basal metabolic pathways under favorable conditions. Dormant bacteria are also resistant to front line antibiotics such as isoniazid which primarily target metabolically active cells.

Attainment of dormant state by *M*.*tb* in the host instigates a state of latent TB infection (LTBI). LTBI can be further defined as a clinical state arising due to the host defense mechanisms whereby the host remains asymptomatic (Singh et al., 2020). There exists different school of thoughts regarding latency and its role in increasing the global TB burden. Further confirmation is required regarding persistence of the bacteria in humans. Several epidemiological studies have investigated the time period of the persisting infection i.e., infection after a span of about 2 years is majorly due to recently transmitted infection (Behr et al., 2019; Behr et al., 2021). The widely used tests for LTBI- the tuberculin skin test and interferon gamma release assay fails to give a clear-cut explanation about the spectrum of disease progression in TB endemic areas (Behr et al., 2018). The limitation to detect LTBI in TB endemic areas can prove fatal in patients undergoing Hematopoietic stem cell transplant (HSCT). Studies have shown that TB is one of the major complications associated with HSCT. The authors from one such prospective cohort study have suggested LTBI screening, detection, and prophylactic treatment with isoniazid (INH) of patients as a safer measure to avoid reactivation of TB after HSCT (de Oliveira Rodrigues et al., 2021).

Persisters refers to a bacterium population with non-heritable phenotypic resistance that arises due to antibiotic pressure through stochastic or deterministic epigenetic factors. Upon removal of antibiotic stress, they can still give rise to antibiotic-sensitive parent populations. The mechanism of persistence is due to survival strategies such as efflux pumps, biofilm formation, altered metabolism, etc. This can even have a role in the emergence of multi-drug resistant strains (Bhaskar et al., 2019).

Models of dormancy can be categorized into two types, *in vitro*, and *in vivo* models. The most used *in vitro* models are the Wayne model, starvation model, nitrous oxide-based model, *in vitro* granuloma model, low oxygen recovery assay, and whole cell nitrate reductase assay. *In vivo* models can be generated using mice, rats, rabbits, guinea pigs, zebrafish, and non-human primates (NHP). Cornell model and low-dose chronic mice infection model are the most widely used murine models for establishing dormancy phenotype. These have been reviewed in detail in various publications along with their limitations (Murphy and Brown, 2007; Alnimr, 2015; Bhaskar et al., 2019).

Singh et al. have proposed a new *ex vivo* dormancy model by using human mesenchymal stem cells (MSCs). Upon using a low multiplicity of infection, *M.tb* survived for 22 days in MSCs without causing host cell death. There was an upregulation in the expression of HspX and increased tolerance to the anti-tubercular drug, isoniazid and rifampicin thus indicating a non-proliferative dormant-like phenotype (Singh et al., 2020).

NHPs are a better option for modeling human TB due to physiological and immunological similarities. In these, macaques such as rhesus and cynomolgus have been widely used (Mothé et al., 2015). Apart from these, the common marmoset (*Callithrix jacchus*) has recently been used (Via et al., 2015). It is susceptible to *M.tb* infection by aerosol and intra-tracheal methods. It demonstrates granulomas closer to humans and survives for greater than 300 days thus can be used for developing a chronic model of infection. Moreover, breeding pairs usually bear twin or triplet offspring allowing for a certain degree of homogeneity across studies (Via et al., 2013). Other *in-vivo* models that are under study for LTBI includes Wistar rat and minipigs and Chinese tree shrew (Gaonkar et al., 2010; Zhan et al., 2014; Rodrigues-Junior et al., 2017).

### 2.1 *Mycobacterial* genes upregulated during dormancy

To unravel the genes involved in dormancy, Iona et al. used the Wayne hypoxia model with forty days of dormancy. Upon studying the kinetics of gene expression during the dormancy period using quantitative real-time PCR (qPCR), they observed upregulation of dormancy regulon genes such as *devR*, alpha-crystallin (*acr*), triglycerol synthase *(tgs1*) in non-replicating persistence stage (NRP-1) after a week of log phase replication. Apart from this, genes involved in general metabolism like *fad26* and nitrate metabolism gene such as *narK2* were also unregulated. After around 11 days, entry of bacterium into the NRP-2 phase was confirmed through methylene decoloration assay. During the transition from NRP-1 to NRP-2, hypoxic genes such as sigma factors (*sigB, sigE, rpoB*), thioredoxin reductase *(trxB1)*, hexose monophosphate shunt pathway *(hmp)*, and *narX* were upregulated. Few genes such as sigma factors involved in transcription (*sigA, sigF, sigH)*, genes involved in general metabolism (Isocitrate lyase*-icl*, Phthiocerol Dimycocerosates*-fadD21*, Heparin-Binding Hemagglutinin Adhesin*- hbhA, fadD26, kasB, lipY*, Cyclopropane synthase gene*-cma2*), genes with miscellaneous functions (Superoxide dismutase-*sodC, relA, mprA*) were constantly expressed throughout forty days of dormancy period suggesting their requirement for survival and basal gene expression. Apart from this, genes such as early secretory target antigen- *esat-6*, Fibronectin binding protein-*fbpB*, culture filtrate protein-*cfp10, dnaA, Ftsz* were downregulated during hypoxic growth phase indicating reduced log phase replication (Iona et al., 2016).

RNA-seq at day 25 of hypoxia establishment revealed the activation of dormancy genes such as *DosRST, MprAB* alternative sigma factors *SigE, SigH*, genes involved in encoding isocitrate lyase (*icl-1)* and methylcitrate lyase (*prpC)*. Authors also observed induction of Caseinolytic protease gene regulator (*ClgR)* regulon including the Psp system in the *SigE* subnetwork which also presumably helps in maintaining membrane integrity under envelope perturbing conditions. The genes involved in uptake and catabolism of lipids were upregulated during persistence phase such as the genes involved in fatty acid beta-oxidation and degradation pathways, genes regulated by *KstR* that controls the expression of a cluster of mycobacterial genes involved in lipid degradation, *Mce* transport systems (*Mce1-4*), which encode putative ABC transporters involved in diverse lipid transportation across the cell wall, genes involved in glyoxylate and dicarboxylate metabolism which is a canonical pathway for lipid utilization (Du et al., 2016).

*M.tb* facing *in vitro* hypoxia in the low stirring sealed tubes was instrumental in elucidating the necessity of *de novo* ATP synthesis to maintain basal ATP level in hypoxic nonreplicating conditions. Anaerobic electron transport chain (ETC) with Type 2 NADH dehydrogenase (*ndh2)* as an initiation enzyme is also functional during hypoxia and is necessary for generating proton motive force (PMF) (Rao et al., 2008).

Microarray profiling of *M.tb* infected murine lungs and broth grown cultures by Talaat et al. has been successful in characterizing differential expressed genes between mice models like Balb/c, SCID and *in vitro* culture at different time points of 7, 14, 21, 28 days post infection. SCID mice with an immune-compromised phenotype showed expression profile like culture grown log phase bacterium. Core *in vivo* regulated genes included- *rubB, dinF*, and Ferredoxin reductase (*fdxA*) which were induced under condition of low pH, DNA damage stress, regardless of host immune status. Few genes were upregulated only in *in vivo* model of Balb/c such as sucrose *proZ* (transport system permease protein), *aceA* (probable isocitrate lyase involved in lipid metabolism), and genes encoding regulatory proteins such as *sigK, sigE*, and *kdpE* (transcriptional regulatory protein). Some genes were distinctly upregulated during *in vitro* growth only such as *cstA* (carbon starvation-induced stress response protein), *cysW* (sulfate transport system permease protein), *Rv3383c* (transferase involved in lipid biosynthesis), and gene-encoding regulatory proteins such as *sigJ* and *Rv1167c* or *Rv1994c* (transcriptional regulators). Transcript analysis of Balb/c at 21 days indicated macrophage activation characterized by low pH and stressors that could lead to a dormant phenotype of the bacilli. In this study *LipF* was identified as one of the important gene for mycobacterial persistence (Talaat et al., 2004). Hence, different transcript profiles were observed among different models of dormancy, suggesting the effect of environmental conditions in influencing bacterial transcription in dormancy regulation.

Another study analyzed the expression of *M.tb* mRNA in C57BL/6 mice model using qPCR and they revealed that the gene expression signature in the granulomatous lesion corresponded to cellular hypoxia, C2 carbon metabolism, and survival in a limited iron environment. Icl, an enzyme of glyoxylate cycle is required for the long-term persistence of *M.tb* in mice indicating a switch to the C2 cycle for long-term persistence. Pyruvate carboxykinase (*pckA*) was also elevated in aerosol-infected mice at 9 weeks post-infection, thus indicating growth on fatty acid substrates. Heat shock protein gene *hspX* which encodes alpha-crystallin with a role in adaptation to stimuli such as hypoxia and nitric oxide is also induced during the stationary growth phase (Timm et al., 2003). The mice model C3HeB/FeJ (Kramnik mice) closely replicates the human granuloma features, as it is hyper-susceptible to *M.tb* infection due to the presence of super susceptibility to tuberculosis 1 (*sst1*) locus (Poh et al., 2022). Research conducted by Harper et al. using the Kramnik mice model showed upregulation of several hypoxia-associated genes such as *dosR/devR, hspX, tgs1*, and *narK2* in bacteria obtained from necrotic lesions. *Rv1733c* which results in a strong T cell response in latently infected individuals also showed about 4-fold upregulation (Harper et al., 2012).

Recent work by Hudock et al. has delineated *M.tb* transcriptome during latent vs active TB using the NHP model. Hypoxic condition *in vivo* was characterized by marked upregulation of genes belonging to the *dosR* regulon, especially in that highly hypoxic areas. Genes belonging to PE/PPE family were expressed more in active TB (ATB) as compared to LTBI. The expression of toxin-antitoxin genes belonging to the *vapBC* family was upregulated within the granulomas. Alternate sigma factors such as *sigF, sigD, sigJ*, *sigI*, *sigB*, *sigK*, and *sigH* were enhanced to suit the changing environment milieu within the granuloma (Hudock et al., 2017).

Transcript profiling of human lung granulomas using resected tissue samples also revealed upregulation of genes such as *icl*, *narX*, *Rv2557*, and *Rv2558* (Fenhalls et al., 2002).

A proteomic study using the Wayne model of dormancy using the SWATH MS technique has helped in quantifying absolute proteome composition and dynamics during dormancy and resuscitation. The proteomic data correlated well with various available transcript-level data about gene modulation during dormancy. *DosR* regulon proteins, proteins with a role in cell wall synthesis such as (alanine dehydrogenase Ald), proteins involved in lipid metabolism (FadE5, DesA1/2, Tgs1/4, and Icl1), as well as the copper stress-related enzymes MymT (copper toxicity protection) and CsoR (copper-sensitive operon repressor) were found to be about 4-folds upregulated during hypoxia-induced dormancy. Various enzymes subnetworks of menaquinone metabolism, cholesterol degradation, fatty acid metabolism, mycolate biosynthesis, branched-chain fatty acid, sulfur compound metabolism, etc. were upregulated during hypoxia. Enzymes involved in the anaerobic electron transport chain such as narX, narK2, ndh2, and cytochrome bd oxidase were also upregulated (Schubert et al., 2015). The summary of genes up-regulated in various *in-vitro* and *in-vivo* models of dormancy have been tabulated and presented in **Table 1**.

**Table 1:** List of genes upregulated during dormancy in various *in-vitro* and *in-vivo* models.

Model	Method of study	Selected genes upregulated during dormancy	Ref
Hypoxia	qPCR	Dormancy regulon gene- *devR, acr, tgs1* *fad26* gene required for cell wall lipid phthioceroldimycocerosate’s (PDIM) synthesis Sigma factors- *sigB, SigE, rpoB*, (*trxB*1)- for antioxidant defense Hexose monophosphate shunt (HMP) for maintaining carbon homeostasis during dormancy *narX* is a nitrate reductase involved in anaerobic respiration.	(Iona et al., 2016)
Hypoxia	RNA-seq	*DosRST, MprAB*- two-component systemalternative sigma factors *SigE, SigH icl-1*- with role in glyoxylate shunt pathway, methylcitrate lyase, KstR regulated genes involved in lipid degradation and cholesterol catabolism *Mce* transport systems: encodes ABC transporters involved in lipid transportation and utilization.	(Du et al., 2016)
Lung from Mice model- *Balb/c* and SCID were compared with invitro grown bacterium under hypoxic conditions	Micro array	Core *in-vivo* regulated genes with role in dormancy were found to be *rubB, dinF*, and *fdxA*. *In-vitro* regulated genes-*cstA* (carbon starvation-induced stress response protein), cysW (sulfate transport system permease protein), *Rv3383c* (transferase involved in lipid biosynthesis), *sigJ* and *Rv1167c* or *Rv1994c* (transcriptional regulators).	(Talaat et al., 2004)
Lung from C57BL/6 mice model	Quantitative real time PCR	*Icl, pckA, hspX*	(Iona et al., 2016)
Non-human primate model (NHP)	Intra- granulomatous gene expression study using transcriptomics	Genes belonging to *dosR* regulon, toxin-antitoxin genes, alternate sigma factors- *sigF, sigD, sigJ, sigI, sigB, sigK*, and *sigH*	(Hudock et al., 2017)
Human lung granuloma	RNA-RNA *in-situ* hybridization	*Icl, narX* *Rv2557* and *Rv2558*- carbon starvation inducible genes *iniB* and *kasA*- upregulation due to isoniazid exposure.	(Fenhalls et al., 2002)

### 2.2 Host-pathogen interactions during dormancy

**Figure 1:**
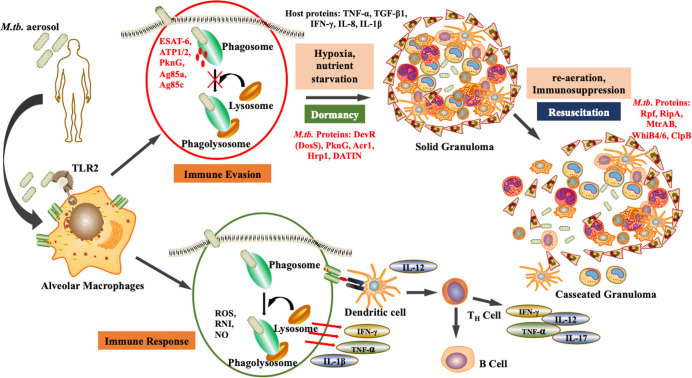
Pathogenesis of *M.tb*: Upon aerosol inhalation, alveolar macrophages degrade bacteria through ROS production and phagolysosomal degradation, which activates other immune cells such as T cells and B cells, further providing defense against the pathogen. However, *M.tb.* adapts the ability to inhibit phagolysosomal degradation, and therefore under unfavorable conditions such as hypoxia undergo dormancy. As the environmental conditions become favorable, the bacteria resuscitate, resulting in active disease. (Red represents *M.tb.* antigens).

After aerosol inhalation, *M.tb* is phagocytosed primarily by alveolar macrophages and resident dendritic cells (Cohen et al., 2018). The bacteria are then killed by phagolysosomes and antigen processing occurs to mediate the immune response. However, *M.tb* is a successful pathogen that has evolved various alternative strategies to survive within the hostile environment of host. *M*.*tb* can escape phagolysosome fusion and utilizes the autophagy pathway to replicate. The maintenance of dormant bacterial state is a dual relationship between the pathogen and the host. The bacteria modulate host factors to ensure successful immune evasion and survival. Upon infection, some bacteria attain non-replicative form and survive in the dormant state within the, granuloma. The granuloma is the inflammation site formed majorly by immune cells such as macrophages NK cells, T cells, B cells, fibroblasts, and neutrophils (Ehlers and Schaible, 2013). These granulomas are heterogenous and the environment within them is skewed towards pro-inflammatory milieu rather than anti-inflammatory. Bacteria persists within the granulomas in extracellular form. *M.tb* replication is obstructed in these sites and thus they are rendered protected from antibiotics. Apart from being a niche for dormant *M.tb*, solid granuloma prevents active disease, the reduced vascularisation restricts dissemination, while caseation results in reactivation as shown in **Figure 1** (Bold and Ernst, 2009). Therefore, during immunosuppression, such as in case of HIV co-infection, there is a caseation of granulomas resulting in reactivation and dissemination of *M.tb* (Pawlowski et al., 2012).

Little is known about the host factors that enable *M.tb* dormancy, yet there are some reports on the host and environmental factors that allow bacteria to become dormant. Most such studies are focussed on *M.tb* and macrophage interaction. The bacteria interfere with the trafficking pathways within the macrophage to inhibit its degradation *via* lysosomal fusion and autophagy (Flynn and Chan, 2003). Macrophages secrete various pro-inflammatory cytokines and chemokines that attract various mononuclear cells including T cells to contain the bacteria within the granuloma. The host factors such as TNF-α and IFN-γ enables the bacteria to survive in acidic conditions, thus providing the aid to the bacteria to reside within the granuloma. Higher TNF-α and apoptosis have been reported to maintain the dormant state of bacteria (Gautam et al., 2014). The macrophage reprogramming is mediated by certain mycobacterial latency proteins such as DosS (DevR), hypoxic response protein 1 (hrp1), latency-associated protein Rv2660c, DATIN, etc., which augments the production of proinflammatory cytokines such as TNF-α, IFN-γ, IL-8, and IL-1β (Kumar et al., 2013; Yihao et al., 2015). There is another study that suggests interaction of *M.tb* with TREM2 receptor of macrophages which reduces antibacterial state in macrophages. This is mediated *via* decrease in TNF-α and ROS levels, enhancing the *M.tb* survival. This is also accompanied by upregulation of anti-inflammatory cytokines such as IFN-β and IL-10 (Iizasa et al., 2021).. The exact mechanism of how these proteins modulate the macrophages to form granuloma is yet to be elucidated. Another study reveals that during dormancy, the alpha-crystallin protein-1 (Acr-1) of *M.tb* provides immunogenic tolerance by inhibiting the maturation of dendritic cells (Siddiqui et al., 2014). In contrast to this, Mubin et al. in 2018 reported that Acr-1 regulates and inhibits the maturation of differentiated macrophages while activating naïve macrophages *via* phosphorylation of STAT-1 and STAT-4. This is accompanied by the generation of *M.tb*-specific T cells that can prevent disease (Mubin et al., 2018). Being a dormancy-associated gene, Acr-1 primes the host immune system to mount an immune response against the bacteria.

*M.tb* has benefitted by exploiting resident mesenchymal stem cells. Unlike macrophages, mesenchymal stem cells (MSCs) provide cytosol as the niche for bacterial replication and rapid lipid synthesis for maintaining dormancy (Fatima et al., 2020). Bacteria live longer in MSCs in a dormant state by hiding themselves in lipid droplets and rendering themselves resistant to combination antibiotics therapy (Fatima et al., 2020) (Jain et al., 2020). It has also been suggested that MSCs are less tolerant to bacterial replication, unlike macrophages, but rather promote dormancy. In turn, *M.tb* induces expression of genes accountable for the quiescent state of MSCs that includes Sox-9, NOTCH-1, and FOXO3a. RNA-seq analysis conferred that autophagy inhibition is one of the strategies employed by the bacteria to maintain a dormant state (Fatima et al., 2020). A recent comparative study suggested that ABCG2 efflux pumps of bone marrow-derived MSCs are upregulated in presence of rifampicin, conferring resistant phenotype to the bacteria. Therefore, targeting these pumps will augment the action of rifampicin (Kaur et al., 2021), however, this observation needs further characterization and analysis.

Yet, there are a lot of other host cells and factors participating in *M.tb* pathogenesis including dormancy and persistence. Host environmental factors such as hypoxia, nutrient deprivation such as depletion of potassium, and availability of Vitamin C are major drivers of the dormancy of the bacteria. It has been stated that Vitamin C (Ascorbic acid) forages oxygen and induces *DevR* regulon to create a hypoxic environment and thereby dormancy (Taneja et al., 2010). Vitamin C promotes growth arrest, while vitamin C removal leads to reactivation of the Viable but non-culturable (VBNC) state of the bacteria. Thus, Vitamin C adjunctive therapy accompanied by anti-TB drugs can help eliminate the disease (Sikri et al., 2018).

### 2.3 BPaL incorporation in MDR treatment regimen

WHO has improvised the treatment regimen for TB known as Direct Observed Treatment Short Course (DOTS) containing the following antibiotics- Isoniazid (H), Rifampicin (R), Ethambutol (E), and Pyrazinamide (Z). As per WHO guidelines 2020, patients with rifampicin resistance or Multi Drug Resistant (MDR) TB are treated, with a combination of drugs which generally include Levofloxacin, Bedaquiline, Linezolid, Clofazimine and Cycloserine. If these drugs cannot be used, then other drugs like Ethambutol, Delamanid, Pyrazinamide, Meropenam, Amikacin, Ethionamide, p-aminosalicylic acid are added to complete the regimen (Andries et al., 2005). For treatment of LTBI, combination (Andries et al., 2005)mbination of Isoniazid, Rifapentine and Rifampin is recommended (World Health Organization, 2020).

According to the latest WHO 2022 guidelines, a new regimen is on a roll out in several countries including India for MDR TB (with resistance Fluoroquinolones) consisting of Bedaquiline, Pretomanid, and Linezolid (BPaL). This drug combination has been found to reduce the treatment regimen from eighteen months to six months (Migliori and Tiberi, 2022). The search for drugs to eliminate latent and persistent bacilli populations is continuing. The recently rolled out BPal regimen can be one of such possible leads in this direction. Studies by Andries et al. proposed the necessity of ATP synthase in *M.tb* survival despite its downregulation as compared to log-state replicating bacilli, thus making it a suitable target for dormant bacteria. Therefore, Bedaquiline which is a diarylquinolone with the ability to target the F0-F1 ATP synthase of the pathogen can target dormant bacilli. Several *in vitro* and *in vivo* models of dormancy have found a significant decline in CFU using this drug (Andries et al., 2005). Pretomanid, a nitroimidazole has also been documented to have positive correlates in the reduction of the bacteria in *in vitro* and *in vivo* dormancy models (Egorova et al., 2021). It is a pro-drug, and is known to release NO within mycobacterial cells. Apart from targeting mycolic acid synthesis, it also results in respiratory poisoning for the bacterium, and NO targets various enzymes in *M.tb* such as ATP synthase, Pks13,and RNA polymerase (Singh et al., 2008; Manjunatha et al., 2009). Linezolid is another antibiotic known to inhibit bacterial protein synthesis by binding to 23S rRNA in the bacterial 50S ribosome. It was found to have potent bactericidal activity against non-replicating bacterial cells in the murine model of latent infection (Zhang et al., 2014). With these promising results on the above-mentioned drugs. WHO has rolled out a new regimen for MDR TB (with resistance Fluoroquinolones) consisting of Bedaquiline, Pretomanid, and Linezolid (BPaL). This drug combination has been found to reduce the treatment regimen from eighteen months to six months (Migliori and Tiberi, 2022).

### 2.4 Drugs and vaccines in evolution against dormant *M.tb*

**A. Drugs:** Several of the drugs currently in use for TB target actively replicating bacteria. There has been a lag in studying and developing drugs that specifically target dormant phase bacteria and this has been a major turn-off for developing effective short-course therapies. The various models – *in vitro* or *in vivo*, which are used to study dormancy fail to fully capture the entire clinical situation of dormancy. In this direction, meta-analysis studies can be quite helpful to predict druggable targets by integrating various results which help in better capture of granuloma heterogeneity (Murphy and Brown, 2007). Few molecules have entered the drug discovery pipeline with efficacy in eliminating the non-replicating form of the bacilli and are in the preliminary stages of study as presented in **Table 2**

**Table 2:** List of drugs under clinical trials trial against dormant *M.tb*.

Drug	Target	Developmental stage	Ref
CPZEN-45	WecA enzyme	Late-stage pre-clinical development	(Takahashi et al., 2013; Zhang et al., 2014)
Nitrobenzothiazinone (BTZ-043)	DprE1	Ia/IIb stage trial	(Ishizaki et al., 2013)
Allylaminomethanone-A, 7-methoxy-2-naphthol	MenA	Lead identification stage	(Makarov et al., 2009)
Salicylanilide esters	ICL	Not entered clinical trial	(Shetye et al., 2020)
HC106A	DosRST	Hit identification stage	(Lee et al., 2015)
PDKA analogues	Malate synthase	Hit identification stage	(Zheng et al., 2017)
(3-(3,4-Dichlorophenyl) ureido) benzoic acid	Cysteine synthase	Hit identification stage	(Schnell et al., 2015)
4-methoxy-2-(pyridin-4-yl) thiazole-5-carboxylic acid	Lysine ϵ-Aminotransferase (LAT)	Hit identification stage	(Betts et al., 2002)

•**CPZEN-45:** It is a nucleoside analog that targets mycobacterial WecA enzyme, which is involved in the synthesis of the arabinogalactan layer of the mycobacterial cell wall and has been found effective against replicating and non-replicating form of the bacteria (Takahashi et al., 2013). It is a derivative of caprazamycin and it is in late-stage preclinical development (Ishizaki et al., 2013).
•**Nitrobenzothiazinone (BTZ-043):** It is a covalent inhibitor of DprE1, an enzyme that is involved in -epimerization of decaprenylphosphoryl ribose (DPR) to decaprenylphosphoryl arabinose (DPA) during arabinan synthesis of the mycobacterial cell wall. Nitrobenzothiazione derivatives have been found to effectively inhibit replicating and non-replicating *M.tb.* It is currently in phase Ia/IIb trial (Makarov et al., 2009).
•**Thioquinazoline (CBR3465), 2-mercapto-quinazolinones:** These are known to inhibit bacterial NADH dehydrogenase-2 (NDH-2), which has a role during both aerobic and anaerobic growth of the bacterium. These are in the lead identification stages.
•**Allylaminomethanone-A, 7-methoxy-2-naphthol:** Menaquinone biosynthesis is an attractive target for dormant stage bacterium. Consequently, inhibitors targeting enzymes, such as MenA of menaquinone biosynthesis are currently in the lead identification stage (Shetye et al., 2020).
•**Salicylanilide esters:** This inhibits ICL, which is found to be upregulated in non-replicating *M.tb*. This inhibitor is currently in the lead identification stage. Despite the importance of ICL in dormant TB infection, no chemical inhibitor is currently in clinical trials (Krátký et al., 2012). The already available small molecule inhibitors such as itaconate, itaconic anhydride, 3-bromopyruvate, oxalate, malate, and 3- nitropropionate (3-NP) are highly cytotoxic (Lee et al., 2015).

Based on data from various models, efforts are underway to synthesize and test small molecule inhibitors specifically targeting dormant and non-replicating bacilli. A few of them are mentioned below-

•**HC106A, 1-(4-fluorophenyl)-3-(isoxazol-5-yl)urea:** These are known to inhibit *DosRST* and were identified through chemical library screening using the *DosRST* regulon fluorescent *M.tb* reporter strain CDC1551 (Zheng et al., 2017).
•**PDKA analogs:** They target malate synthase which is involved in the second step of the glyoxylate shunt pathway after the ICL step, and thereby inducing dormancy. Krieger et al. have synthesized methyl ester prodrug of (Z)-2-hydroxy-4-oxo-4-phenyl but-2-enoic acid (PDKA) analogs (Krieger et al., 2012).
•**(3-(3,4-Dichlorophenyl) ureido) benzoic acid:** Cysteine is known to play role in mycothiol biosynthesis, which helps in maintaining redox balance during *M.tb* dormancy. Cysteine synthase is an enzyme involved in the synthesis of cysteine using O-phosphoserine and a sulfur carrier protein CysO (Schnell et al., 2015). Urea-based compounds such as 3-(3-(3,4-Dichlorophenyl) ureido) benzoic acid have been identified as hit compounds to target cysteine synthase by Brunner et al. by using a nutrient starved model of *M.tb* dormancy (Brunner et al., 2016).
•**4-methoxy-2-(pyridin-4-yl) thiazole-5-carboxylic acid:** Lysine ϵ-Aminotransferase (LAT) enzyme has been found to have a role in persister population formation in mycobacterium (Duan et al., 2016). Studies by Betts et al., have shown this enzyme to be highly upregulated during nutrient starved model of dormancy (Betts et al., 2002). Sriram et al., have identified several hits against this target such as 4-methoxy-2-(pyridin-4-yl) thiazole-5-carboxylic acid, 1-((4-methoxyphenyl) sulfonyl)-40,50 -dihydrospiro[piperidine-4,70 -thieno[2,3-c]pyran], 2,20-oxybis(N0-(4-fluorobenzylidene) acetohydrazide). These compounds have shown positive results in *in-vitro* dormancy model (Devi et al., 2015).
•Several compounds such as quinoxaline-based molecule (6-(4-(4-fluorophenyl)piperazin-1-yl)-3-(methoxycarbonyl)-2-(trifluoromethyl)quinoxaline 1,4-dioxide), 3-triazenoindole-based molecule TU112(ethyl ester of 3-((4-methylpiperazin-1-yl) diazenyl)-1H-indole-2-carboxylic acid), thiazole derivative (ethyl ester of 2-cyclohexyl-5-(quinoxalin-6-yl)thiazole-4-carboxylic acid; compound), benzimidazole–acrylonitrile hybrid (2-(1H-benzo[d]imidazol-2-yl)-3-(4-(4-(ptolyl)piperazin-1-yl)phenyl)acrylonitrile), pyrano [3,2-b] indolones (2-oxo-2,5-dihydropyrano[3,2-b]indole-3-carbonitrile), PAMCHD (2-(((2-hydroxyphenyl)amino)methylene)-5,5-dimethylcyclohexane-1,3-dione) have been synthesized or identified as hit compounds by several research groups with proven effectiveness inhibiting dormant mycobacterium in *in-vitro* studies. But further research is required to convert these molecules into the lead compounds and test their efficacy in *in-vivo* models, and to elucidate the mechanism of action and target sites of these compounds (Egorova et al., 2021).

**B. Vaccines:** BCG, the only approved vaccine currently in use for TB fails to provide long-term immunity in adults. The waning immunity in the long term is due to failure in the generation of memory T cell response, and inadequate response to latency-associated antigens. BCG only generates cell-mediated immunity to secretory antigens which are mainly expressed by the rapidly growing bacterium (Dey et al., 2011). The deleted RD1 region of BCG contains several genes that are upregulated during hypoxia and nutrient-starved model of *in vitro* dormancy; thus BCG vaccine, does not prevent the establishment of latent TB infection (Andersen, 2007). Since the maximum population is vaccinated with BCG, vaccination strategies that can complement it and improve the long-term immunological response are being searched for. In this direction, researchers have proposed vaccine strategies such as the first stage use of BCG vaccine followed by second-stage use of vaccine several years after BCG such that this vaccine can act as BCG prime-boost and boost memory responses. A third stage post-exposure vaccine can be administered to prevent reactivation of latent TB (Ottenhoff and Kaufmann, 2012; Weiner and Kaufmann, 2014).

At present several prophylactic subunit vaccines are in clinical trial against TB as tabulated in **Table 3**, but they are based on antigens secreted by actively replicating bacilli thus undermining their utility against reactivation of LTBI (Khademi et al., 2018). Two subunit vaccines have used dormancy antigens and are in phase 1 of clinical trials. These vaccines are H56 + IC31 (H56 is a subunit vaccine candidate formulated with TLR9-agonist cationic adjuvant IC31)and ID93 + GLA-SE (ID93 is a subunit vaccine candidate with TLR4-agonist adjuvant known as Glucopyranosyl Lipid A [GLA] in a nano emulsion formulation), and have been found to generate multifunctional CD4^+^T cell response, strong Th1 response, and limit reactivation of latent TB in animal models (Weiner and Kaufmann, 2014; Luabeya et al., 2015; Khademi et al., 2018; The TBVPX-113 Study Team et al., 2018; Kaufmann, 2020).

**Table 3:** List of vaccines currently under study against dormant *M.tb*.

Vaccine	Vaccine type	Ref
H56 + IC31 and ID93 + GLA-SE	Prophylactic subunit vaccines	(Andersen, 2007; Ottenhoff and Kaufmann, 2012)
Rv3131	Antigen for multi-antigenic subunit vaccine	(Lu et al., 2022)
AEC/BC02	Subunit vaccine	(Rai et al., 2018)
L91	Lipidated multistage epitope-based vaccine	(Khademi et al., 2018)
Latency antigens incorporated in Modified ankara virus vector	Multi-antigenic, multiphasic vaccine	(Kwon et al., 2017)
RUTI	Therapeutic vaccine	(Cardona, 2006; Leung-Theung-Long et al., 2015)

Kwon et al. have identified Rv3131, a nitro reductase, as a suitable antigen for use in multi-antigenic subunit vaccines. This antigen was found to be upregulated during exponential as well as hypoxic growth conditions. Rv3131 administration along with GLA-SE which is a TLR4 adjuvant displayed a significant reduction in bacterial number, less extensive lung inflammation, improved multifunctional and specific CD4^+^ T cells, effector/memory T cell expansion, improved serum IgG2c response in mice model (Kwon et al., 2017).

Subunit vaccine AEC/BC02 developed by. Lu J et al., has been found effective in reducing tissue lesions and providing protection in latently *M.tb* infected mice. The vaccine candidate also inhibited *M.tb* infection in latently infected guinea pigs. Th1-mediated immune response was predominately observed. This vaccine candidate can be further researched as a post-exposure candidate for latent *M.tb* infection (Lu et al., 2022).

Rai et al., have designed a lipidated multistage epitope-based vaccine L91 which comprises a promiscuous CD4^+^ T cell epitope of latently associated Acr1 antigen of *M.tb* and TLR-2 agonist Pam2Cys. This candidate showed better antigen processing and presentation by DC, improved release of IFN-γ by peptide-specific CD4^+^ T cells, and generated significant memory T cell responses. L91 immunization had better protection than BCG in mice, guinea pig, and PBMC derived from PPD^+^ healthy volunteers. This construct was further refined using CD8^+^ T cell epitope from *M.tb* antigen (TB10.4) and renamed as L4.8 (Rai et al., 2018).

Leung-Theung et al. have designed a multi-antigenic, multiphasic vaccine based on the modified Ankara virus. This construct contained 14 antigens representative of the active, latent and resuscitation phases of TB. The 3 protein fusions used were - RpfB-RpfD-Ag85B-TB10.4-ESAT-6, SF-Rv2029-Rv2626-Rv1733-Rv0111(SF- signal peptide of measles virus), and SR-Rv0569-Rv1813-Rv3407-Rv3478-Rv1807-TMR (SR-glycoprotein precursor of rabies ERA strain, TMR-membrane anchoring peptide from rabies glycoprotein). This construct was found effective in the generation of CD4^+^T cell response, CD8^+^T cell response, and effective cytokine generation in mice and non-human primate models (Leung-Theung-Long et al., 2015).

Therapeutic vaccine such as RUTI is currently under phase 1 clinical trial. It is a semi-purified and detoxified fragment of *M.tb* that had been cultured under stress and starvation conditions that can result in the enrichment of dormancy antigens (Cardona, 2006; Vilaplana et al., 2010).

## 3 Resuscitation of dormant *M.tb*

Depending upon several genetic and environmental factors, the latent bacteria are capable of undergoing reactivation or resuscitation leading to a clinically characterized state. Clinically known as resuscitation, reactivation is the active phenotype of *M.tb* after exiting the dormancy and may take up to months or years to happen. Of those latently infected with TB, about 10% of individuals re-develop the disease and are clinically diagnosed with active disease. This state is determined by the ability of the bacteria to re-grow and multiply conferring active disease upon forbearing conditions. Coinfections like HIV, metabolic disorders like diabetes or other drug-induced immune suppression as in organ transplant patients are the major drivers of resuscitation.

Resuscitation-promoting factors (Rpfs) are secreted by Actinobacteria and were initially identified in *Micrococcus luteus* (Mukamolova et al., 2002). These factors are indispensable for exiting dormancy but are not crucial for bacterial growth (Wang et al., 2021). In *M.tb*, there are five factors- rpfA-E, sharing a conserved domain known as Domains of Unknown Functions (DUF) and while RpfB and RpfE have G5 domains in addition to DUF that bind some cytoplasmic proteins. The distinguishing feature of *M.tb* rpfs is the presence of the lysozyme C and lytic transglycolase domain, possibly important in cell wall metabolism. Researchers have shown that rpfs show their catalytic activity in hydrolyzing peptidoglycan. This implies that rpfs comprise enzymatic features that allow the transition of bacteria from dormancy to reactivation (Kana and Mizrahi, 2010). There have been numerous attempts to find the other factors that participate in the resuscitation process. To date, only one factor known as the Resuscitation-promoting factor interacting protein (RipA), is discovered that interacts with RpfB to allow cell division. These two factors are known to act synergistically to modify the peptidoglycan at specific regions, thus exiting the dormancy (Nikitushkin et al., 2015). It is also reported that the deletion of protein kinase G (*pknG*) boosted the bactericidal activity of anti-TB drugs and subsequently attenuated the ability of the bacteria to resuscitate in antibiotic treated mice, thus contributing as one of the important adjunct therapy to prevent resuscitation (Khan and Nandicoori, 2021). However, there should be other supporting bacterial factors that interact with Rpfs to allow the transition to resuscitation, therefore, this area of research should be explored.

It is worth acknowledging how RpfB is functional in dormant cells to promote reactivation. Therefore, some studies have explored the bacterial components and have identified the role of one of the essential two-component systems in the regulation of RpfB. These include the MtrAB two-component system that regulates the transcription control of RpfB. MtrA is a response regulator that represses the expression of *rpfB* by binding to the promoter region of *rpfb* (Sharma et al., 2015). Apart from MtrAB, the balance of RpfB activity is maintained by co-regulation of cell wall synthesis, ribosome maturation, and resuscitation, preventing cellular toxicity. This regulation is performed by the RNA switch that permits co-transcription of *ksgA*, a methyltransferase that plays a crucial role in ribosome maturation, and a kinase, *ispE* important in cell wall synthesis. This is essential for the efficient protein synthesis, thus easing in transition to resuscitation (Schwenk et al., 2018). Hence, the process of resuscitation is under multidimensional control involving early and co-transcriptional regulation but a detailed investigation is further required.

### 3.1 Transcriptional regulation

It is believed that during different phases of *M. tb* replication, dormancy and resuscitation, there are alterations in the transcriptional and translational state of the bacteria. Minimal efforts are put to identify such changes and reports have shown that there is an immediate transcriptomic burst of coding and non-coding RNA during resuscitation. During dormancy, there is less lipid content in bacterial cells as compared to replicating cells (Gengenbacher and Kaufmann, 2012). Hence to combat the stress, bacteria tend to upregulate the fatty acid synthase, ensuring the higher synthesis of mycolic acid that assist in cell wall synthesis for replication (Choi et al., 2000). Another major upregulation was observed in the expression of heat shock proteins and endopeptidase ClpB, which plays a crucial role in degrading misfolded proteins and maintaining protein turnover, thereby increasing the probability of reactivation (Sherrid et al., 2010). To support the growth, there is an increase in the expression of genes involved in metabolic pathways to allow cell division. WhiB6 is a transcriptional regulator that controls the metabolism and virulence of *M.tb* and is a dormancy related factor (Chen et al., 2016). These genes are involved in the early stages of resuscitation, yet some genes are more upregulated in the late stages of reactivation. These included genes responsible for aerobic respiration, ATP synthesis, and the TCA cycle to meet the energy demands of the cell to replicate (Salina et al., 2014). Iona et al. (2016), reported that several genes were upregulated during resuscitation similarly to the exponential phase (Salina et al., 2019).

### 3.2 Translational regulation

Despite the upregulation in transcription, minimal efforts have been made to identify the changes at the proteome level during resuscitation. SWATH-MS is a modified technique that provides the advantage of absolute quantification of the proteome in a cell. Through this technique, the absolute protein concentration of the whole cell lysate was estimated. It was observed that intracellular levels of ATP were significantly higher during the exponential phase, gradually decreasing upon hypoxia-stress i.e., dormancy. However, after re-aeration, ATP levels increased but were not equivalent to the exponential phase. Despite differences in ATP levels, F0-F1 ATP synthase levels were unaffected while other enzymes involved in energy production and metabolism were significantly upregulated. For example, NADH dehydrogenase I/II, cytochrome c reductase, cytochrome b oxidase, cytochrome c oxidase, and nitrate reductase were downregulated during resuscitation as compared to dormancy. Also, it is worth noting that enzymes involved in the methylcitrate cycle were present in higher copies during 2 days after re-aeration, suggesting that these enzymes are involved in the early phase of resuscitation. These enzymes were PrpC and PrpD. Other proteins were also upregulated during dormancy that included sigma factors such as SigE and Sig B, as well as the transcriptional regulator ClgR that regulates ClpP (Gopinath et al., 2015) (Schubert et al., 2015). However, the proteome experiments were performed on *in vitro* cultures. Since the host environment is crucial to the growth of bacteria, *in vivo* proteome study is essential and thus should be explored.

There are still missing gaps that are required to be filled to comprehend the mechanism of resuscitation. Understanding the minute changes at the transcriptional and translational levels will assist in designing the therapies against reactivation, preventing disease transmission.

### 3.3 Host factors responsible for resuscitation

Progression of *M.tb* in the host is mediated by various immune processes including innate and adaptive arms of the immune system. As discussed earlier, the bacteria hijack the host system to hide in latent form and thus is an escape mechanism of the pathogen from the immune system. Immune cells provide the niche for the extracellular bacteria to be contained in the granuloma. TNF-α is one of the host factors that enable the containment of bacteria in granulomas (Mezouar et al., 2019). However, due to certain environmental and genetic factors, and co-morbidities, suppression of the immune system leads to the dissemination of the bacteria resulting in disease reactivation. Upon resuscitation, the immune system is re-stimulated and generates strong T- cell responses. Researchers have put efforts to understand the antigenic stimulation of such T- cells and observed that Rpfs carry the antigenic determinants triggering the immune system in LTBI. Mutants of Rpfs have been shown to induce greater Th1 immune response and a significant decrease in bacterial load and lung pathology (Kim et al., 2013). Anti- TNF-α induces resuscitation of the bacteria and results in an increase in bacterial load. These TNF-α antagonists such as Adalimumab induce TGF-β1 to promote resuscitation, whereas lymphotoxin-α neutralization prevents reactivation (Arbués et al., 2020). DOTS therapy antibiotics such as isoniazid, rifampicin, pyrazinamide and ethambutol leads to reactivation of the dormant bacteria as it damps the immune system causing decrease in CD4^+^T cells (Skakun and Shman’ko, 1985; Younossian et al., 2005; Singh et al., 2021).

Despite the existing data, this is unclear how host proteins and environment support bacterial reactivation and growth. Therefore, it is one of the fascinating research areas to identify the host factors promoting resuscitation and the immune response toward resuscitated bacteria.

### 3.4 Existing strategies and prospects to combat resuscitation

Understanding the transition from dormancy to resuscitation is key to developing strategies to combat all the phases of disease transmission. Today’s existing knowledge is less sufficient to devise a better therapy against TB reactivation, yet researchers have put forward to bring diverse ways of battling the disease.

**Vaccines:** There are several shreds of evidence showing that Rpf is a vital therapeutic target that has been studied to date. Few antigenic determinants of RpfD have been shown to induce polyfunctional CD4^+^T cells while memory and effector subsets of CD8^+^T cells were also observed (Arroyo et al., 2016). One of the mutants of Rpf is Rpfd2 which can serve as a potential vaccine target due to its ability to induce a higher Th1 response (Kim et al., 2013). Also, of all the Rpfs tested, RpfB has been a promising candidate for the DNA vaccine. It has been shown to induce a modest but significant cellular immune response against TB with higher levels of IL-2 and IFN-γ (Romano et al., 2012). Fan et al., in 2010, have shown that the RpfB domain has the ability to induce humoral response, and thereby, monoclonal antibodies against rpfs may inhibit TB recurrence (Fan et al., 2010). Collectively, Rpfs are suggested to be novel vaccine candidates but require further characterization and analysis to serve as a sub-unit vaccine. It is well-known that only available vaccine against TB is BCG that fails to induce long term memory and thereby insufficient to prevent resuscitation (Negi et al., 2022). Therefore, a potential adjunct complex, termed as peptide-TLR agonist-liposome (PTL) when administered with BCG enhances the long-term memory and reduces the rate of reactivation, thereby reduction in bacterial burden in the lungs of mice (Kumar et al., 2021).

**Inhibitors:** The biological activity of RpfB is dependent on LysM and LytM domains (Sexton et al., 2020), therefore inhibition of Rpf is the potential way to prevent resuscitation and thereby transmission. Further, deletion of *pknG* along with anti-TB drugs has been shown to inhibit resuscitation, suggesting that it can be targeted to prevent resuscitation (Khan and Nandicoori, 2021). Inhibition of TNF-α and lymphotoxin-α with Adalimumab and Etanercept, respectively, have been shown to induce resuscitation *via* TGF-β1 (Arbués et al., 2020). Therefore, suppressing TGF-β1 may show inhibition in resuscitation. On the other hand, computational approaches are utilised to identify small molecule inhibitors such as phytochemicals to inhibit rpfs and thereby resuscitation (Dwivedi et al., 2020). Bergenin is one of the phytochemicals that has been experimentally validated to show the modulation of the existing TB therapy and reduces immune suppression. Thus, bergenin acts as an adjunct to prevent resuscitation or reactivation of *M.tb* (Kumar et al., 2019). Curcumin is another bioactive molecule which when formulated in nanoparticles and administered as adjunct therapy prevents reactivation of the dormant bacteria (Tousif et al., 2017). Similarly, Luteolin has also been shown to provide sterile immunity when given as an adjunct to DOTS therapy and prevents reactivation (Singh et al., 2021).

It is worth noting that inhibitors preventing resuscitation may serve as an important therapeutic intervention to restrict the bacteria in dormant state. This is because it has been known that LTBI can reactivate within 2-5 years of infection (Behr et al., 2018). Thus, sustaining the bacteria in a dormant state would prevent TB transmission, thereby reducing the disease burden globally.

Since the existing anti-TB drugs are ineffective against LTBI, and as mentioned earlier, they are proven to dampen the immune system, therefore, combining inhibitors of resuscitation along with standard anti-TB drugs can prevent the further reactivation and progression of the disease.

**Repurposed drugs**: The repurposing of drugs has proven to be highly efficient in preventing pulmonary TB and thereby, reactivation. One such drug is statins, inhibitors of HMG-CoA reductase. It is known that *M.tb* utilizes cholesterol to infect the immune cells and therefore survive within them (Istvan and Deisenhofer, 2001). Therefore, statins are used as host-directed therapy. It is given as an adjunct with anti-TB drugs that depletes the cholesterol, making environmental conditions harsh for the bacteria survival (Lobato et al., 2014). Similarly, anti-diabetic drug, metformin is also administered along with statins to reduce the prevalence of LTBI and thus, prevents resuscitation (Magee et al., 2019). Along with this, corticosteroids such as dexamethasone is also used an adjunct with standard TB drugs. Dexamethasone inhibits the *M.tb*- induced necrotic cell death in the host thus preventing disease reactivation (Gräb et al., 2019).

Taken together, more understanding of the mechanism of transition from dormancy to resuscitation is required to invent therapies against TB. Also, there are possible host factors that promote reactivation, thus discovering them and modulating them is essential to have definitive host-directed strategies to combat this highly infectious disease.

## 4 Concluding remarks

Of all the infected individuals, 90% of individuals develop LTBI while in 10% of them, the bacteria resuscitate and develop active disease. Thus, controlling the infection at the state of dormancy or resuscitation is important to prevent transmission. Nonetheless, the mechanism of transition from dormancy to resuscitation is less explored due to the lack of a perfect model of dormancy, hence it is a drawback in devising prophylactic and therapeutic strategies. In this direction, bioinformatic studies such as meta-analysis can be a powerful tool to integrate and score results arising from different models of dormancy and thus bridge the limitations prevailing amongst the current dormancy models. Resolving the dilemma over LTBI i.e., whether lifelong latency is associated with TB or it is majorly a transmissible disease from active TB patients can be the key towards the WHO End TB goal. Epidemiological studies and newer biomarker discoveries can help attain this goal by directing resources towards the most definitive cause and eventually reduction in the global TB burden.

Targeting the host factors to battle the disease may provide a better way of preventing disease as compared to the drugs against bacterial factors due to the high chances of emergence of drug resistance. Targeting the host factors as discussed in this review, such as factors responsible for a hypoxic environment that drives granuloma formation seems to be crucial in eradicating the bacteria. Thus, accompanying anti-TB drugs with host-directed therapies can be one of the crucial strategies to prevent TB. Employing combinatorial therapies is the key to fight with drug-resistant TB.

## Author contributions

AB designed the theme of the manuscript. AV and AG conducted the literature search and wrote the manuscript. AV drew the schematic diagram and AG made the tables. AB and VD critically reviewed and edited the final version of the manuscript. All authors read and approved the final manuscript.
